# Harmless Acute Pancreatitis Negative among Cases of Acute Pancreatitis in a Tertiary Care Centre: A Descriptive Cross-sectional Study

**DOI:** 10.31729/jnma.6627

**Published:** 2021-12-31

**Authors:** Ravi Acharya, Peeyush Dahal, Santoshi Parajuli

**Affiliations:** 1 Department of General Surgery, Rapti Academy of Health Sciences, Dang, Nepal; 2 Burn And Plastic Unit, Bir Hospital, Kathmandu, Nepal; 3 Department of Radiology, Bir Hospital, Kathmandu, Nepal

**Keywords:** *acute pancreatitis*, *score*, *severity*

## Abstract

**Introduction:**

Acute Pancreatitis is a common disease, diagnosed in about 3% of cases presenting with abdominal pain. Severe disease with multiple systemic complications develops in 10-20% of the cases which require intensive care in specialized centres. Harmless acute pancreatitis score is a simple and economical score predicting the non-severe course of disease within 30 minutes of admission. The aim of our study was to find the prevalence of harmless (harmless acute pancreatitis) among cases of acute pancreatitis in a tertiary care centre.

**Methods:**

A descriptive cross-sectional study was conducted after obtaining the ethical approval (Reference no. 344/2076/77). The study was carried out from September 2019 to February 2020 taking 50 patients with the first attack of acute pancreatitis. Convenient sampling was done. Harmless acute pancreatitis score prediction of severe disease and final outcome as severe or non-severe was noted with predefined severity criteria. Data were entered in Microsoft Excel and Statistical Package for the Social Sciences and results represented in tables and charts. Point estimate at 95% was done and frequency and percentage were calculated.

**Results:**

Out of 50 patients with first attack of acute pancreatitis, using the harmless acute pancreatitis score, the prevalence of harmless acute pancreatitis was 22 (56%) (44.45-67.5 at 90% Confidence Interval).

**Conclusions:**

The harmless acute pancreatitis score is an easy, less expensive, quick and promising early scoring system for prediction of non-severe courses of acute pancreatitis. The prevalence of harmless (harmless acute pancreatitis) among cases of acute pancreatitis was found to be similar to other studies.

## INTRODUCTION

Acute pancreatitis (AP) is an inflammatory disease with estimated incidence about 3% of cases presenting with abdominal pain.^1,2^ Most episodes of AP are self-limiting and about 20% of patients develop a severe disease with local and extra-pancreatic complications with mortality ranging from 20-60% in severe acute pancreatitis (SAP).^3-5,6^ Wide variety of clinical parameters, single biochemical markers, and imaging procedures for predicting SAP have been developed.^5,7^ But they are either insufficiently sensitive or too complicated, expensive, not readily available or unavailable at all outside specialized centers.^8,9^

Harmless Acute Pancreatitis Score (HAPS), is a simple scoring system with high specificity and positive predictive value, minimizing referral and admission of large proportions of patients who do not require admission or Intensive Care Unit (ICU) care.^8,10^ This can be utilized in triaging cases of AP even in setting where only basic laboratory services are available and helps in reducing the economic burden.

The aim of our study was to find the prevalence of harmless acute pancreatitis negative (HAPS) outcomes of acute pancreatitis using the harmless acute pancreatitis score.

## METHODS

This was a descriptive cross-sectional study carried out in Bir Hospital, Kathmandu, Nepal from September 2019-February 2020 among 50 patients with the first attack of acute pancreatitis. Data collection was started after obtaining an approval from the Institutional Review Board (reference number: 344/2076/77) and informed written consent was taken from each patient.

The sample size was calculated using the following formula,

n = Z^2^ × p × q / e^2^

  = (1.645)^2^ × 0.03 × (1-0.03) / (0.05)^2^

  = 31.48

  = 31

Where,

n= required sample size
Z= 1.645 at 90% Confidence Interval (CI)
p= prevalence taken for maximum required sample size, 50%
q= 1-p
e= margin of error, 5%

Fifty cases participated in our study. Convenience sampling was used to collect the data.

All patients older than 16 years presenting with the first attack of AP were included in the study. Patients with Acute Pancreatitis were diagnosed with two of the following three features (as per the revised Atlanta Classification, 2012).^11^

Abdominal pain consistent with acute pancreatitis (acute onset of a persistent, severe epigastric pain often radiating to the back).

Serum lipase activity (or amylase activity) at least three times greater than the upper limit of normal.

Characteristic findings of acute pancreatitis on contrast-enhanced computed tomography (CECT) and less commonly magnetic resonance imaging (MRI) or transabdominal ultrasonography (USG).

Patients with recurrent AP, acute on chronic AP, known chronic kidney disease, COPD, malignancy, hematologic disorder were excluded from the study. Data, including age, sex, and etiological factors were recorded for each patient in preformed proforma. All the investigations required were routine investigations done in all the cases of Acute Pancreatitis. The etiology of acute pancreatitis was considered to be biliary if stones detected in the gallbladder and/or common bile duct and of alcoholic etiology if the patient or his/her relatives reports consumption of more than 60gms of pure alcohol/day for more than five years. Others identified like endoscopic procedures (endoscopic retrograde cholangiopancreatography with or without sphincterotomy), hyperlipidemia, trauma and drugs were labeled as others. In the remaining cases, the etiology was classified as unknown or idiopathic.

All laboratory investigations were performed in the hospital. CECT abdomen and Pelvis was done on the fourth day of admission. Harmless (HAPS-) course predicting non severe disease was predicted if there was absence of rebound abdominal tenderness or guarding, serum creatinine of <2mg/dL, and hematocrit of <43 for male and <39.6 for female patients at the time of admission. Not Harmless (HAPS+) course was predicted if any of the three parameters was present. Rebound abdominal tenderness was recorded after e valuation by a s urgical on Duty Res ident. Enrolled patients were followed till their hospital stay or death and the final outcome of the patient was as recorded severe or non-severe. Severe acute pancreatitis was defined as the occurrence of as the occurrence of pancreatic necrosis (verified by contrast-enhanced CT scan and a Balthazar score more than or equal to 5),^12^ or need for respiratory or circulatory support or dialysis and/or mortality during the hospital stay.

Data obtained were entered into the computer using Microsoft Excel and IBM Statistical Package for the Social Sciences (SPSS) and results represented in tables and charts. The obtained results were discussed with reference to current world literatures and conclusion was drawn based on these results. Point estimate at 95% was done and frequency and percentage were calculated.

## RESULTS

HAPS prediction of Not Harmless (HAPS+) course of disease was 22 (56%) (44.45-67.5 at 90% Confidence Interval). Out of 50 patients, 30 (57%) were male and 20 (43%) were female. Median days of admission were 4 (1-26) days. The demographic data and clinical characteristics of the study population are enlisted in (Table 1).

**Table 1:** Clinical characteristics of the study cohort and HAPS severity.

Variables	Not Harmless (HAPS+) n (%)	Harmless (HAPS-) n (%)
Median Age (Range) (years)	41.50 (20-70)	38 (18-80)
Male n (%)	15 (68.2)	15 (53.6)
Median Duration of symptoms (Range) (Hours)	34 (12-120)	24 (8-72)
Epigastric Guarding/Rebound Tenderness Present n (%)	7 (31.8)	1 (3.5)
Median Hematocrit (Range)(%)	44.2 (37.8-51.2)	38.85 (25.8-46.2)
Median Blood Urea (Range) (mg/dl)	29.5 (11-291)	20 (12-62)
Median Serum Creatinine (Range) (mg/dl)	0.65 (0.4-4.5)	0.60 (0.4-1.7)
Median Serum Amylase (Range) (IU/L)	445 (84-1846)	636 (97-2950)
Median Serum Lipase (Range) (IU/L)	879 (360-2706)	841.50 (420-5850)
**Etiology**		
Alcohol	12 (54.54)	11 (39.2)
Biliary	6 (27.27)	16 (57.1)
Idiopathic	2 (9.09)	1 (3.5)
Others	2 (9.09)	0

**Figure 1:**
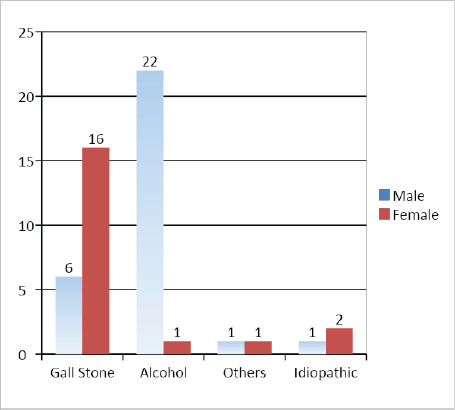
Etiology and gender distribution of patients with acute pancreatitis.

Gender wise etiological distribution is depicted in (Figure 1).

Harmless (HAPS-) course of disease was 28 (44%). Seven (31.8%) patients in the Not Harmless (HAPS + ) group and 1 (3.5%) in the Harmless (HAPS-) group had epigastric guarding or rebound tenderness. Similarly, median hematocrit was 44.2% (37.8-51.2) in HAPS + cases and 38.85 (25.8-46.2) in HAPS-cases. Median serum creatinine was 0.65 (0.45-4.5) mg/dl in HAPS + patients and 0.60 (0.4-1.7)mg/dl in HAPS-patients.

**Figure 2:**
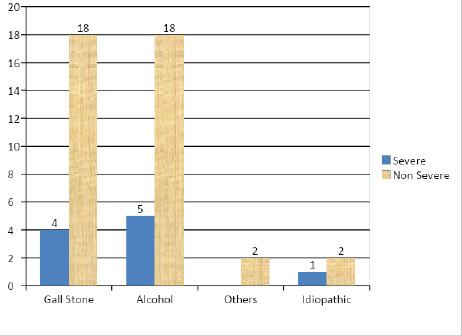
Severity according to etiology of acute pancreatitis.

About 10 (20%) patients had a severe course and 40 (80%) patients had a non-severe course. Out of 28 (44%) patients predicted to have Harmless course 1 (3.57%) had a severe course and among 22 (56%) cases predicted to have a Not Harmless course and 9 (40.9%) had a severe course (Table 2). Severity of disease was not different in biliary or non-biliary Pancreatitis (Figure 2).

**Table 2:** HAPS prediction and severity Outcome.

Outcome Severity HAPS Prediction	Non-Severe	Severe	Total
Harmless ( HAPS -)	27 (96.4)	1 (3.57)	28 (56)
Not Harmless (HAPS + )	13 (59)	9 (40.9)	22 (44)
**Total**	40 (80)	10 (20)	50 (100)

Of the parameters for severe disease, 1 (2%) patient needed Respiratory Support, 2 (4%) needed Circulatory, one (2%) needed Dialysis. There were 4 (8%) patients with pancreatic necrosis (CTSI >5). And there were 2 (4%) acute pancreatitis related mortality.

## DISCUSSION

Acute pancreatitis is an inflammatory process of the pancreas with a wide spectrum of severity, complications and outcome.^13^ The course of the disease is unpredictable, some resolve with appropriate management or may lead to complications which may end in fatality with the overall mortality ranging from 2 to 22%.^14^ Early prediction of such complications at the preventable help reducing morbidity and mortality in such patients.^15^

Lankisch PG, et al. in 2009,^8^ based on the results from a prospectively followed cohort of 394 patients with the first attack of AP who presented at the Department of Internal Medicine in the Luneburg Municipal Clinic, Germany devised this scoring system and found that HAPS could predict a non-severe course with a specificity of 97% (89-99 %) and PPV of 98% (92-100%) in these patients, and this was validated in another validation set of 452 patients in a German multicentre setting, where the predictive accuracy was found to be similar. Further validation came more recently, in 2011, from a Swedish cohort of 511 patients, by Oskarsson V, et al. where HAPS could predict a non-severe disease course with a specificity of 96.3 (81.0-99.9) and PPV of 98.7 (93.1-100).^10^ We found one severe course of disease among 28 predicted among 50 patients to have a non-severe course that is comparable with the study of Lankisch, et al. with large study population.

Another study done at a tertiary centre in India by Talukdar R, et al. with cohort of 103 patients found the Sensitivity, specificity, positive and negative predictive value, and receiver operating characteristics area under the curve of HAPS as a predictor of non-severe disease to be 76.3 (66.9-86.4)%, 85.7 (78.0-96.8)%, 93.8 (88.5-98.6)%, 56.6 (45.4-73.6)%, and 84.8 (76.9-92.7)% respectively.^16^ The results were similar as obtained in our present study. Study population in study by Talukdar R, et al. and our study are from Indian subcontinent and have almost similar characteristics with our study population.^16^

In study by Al-Qahtani HH, et al. conducted in King Saud Medical City, Riyadh, Kingdom of Saudi Arabia, between January 2012 and December 2015 with 116 patients of AP found that HAPS correctly predicted the disease severity in 101 (87%) patients with sensitivity of 98% specificity of 77% and accuracy of 96%.^17^ In contrast, HAPS correctly predicted the non-severe course in 96% in our present study.

Similar study by Jan N, et al. from 06-04-2011 to 1203-2013, at Gastroenterology Unit Hayatabad Medical Complex (HMC), with cohort of 36 males & 48 females, with age distribution from 15 to 65 years, HAPS score initially identified that 72 patients will follow mild course, but later on two patients from this category followed severe course comparable with our study.^18^

In a Turkish study by Sayrac AV, et al.^19^ in a tertiary care university hospital with 144 patients of AP, compared HAPS with Ranson's scores for predicting course of Acute Pancreatitis. The specificity and positive predictive value (PPV) of HAPS were 81% and 96%, respectively, and the odds ratio was 5.57 (1.5120.50) showing comparable results with our study.

Ma X, et al.^20^ studied 703 consecutive AP patients admitted to West China Hospital between January, 2016 and August, 2017, among them 182 were predicted to have harmless AP and 521 to have non-harmless AP, and the patients in the latter group had significantly worse clinical outcomes (P <0.001). The specificity, the sensitivity, the PPV and NPV of HAPS on admission for predicting MAP was 97.7% (95% CI: 95.4-99.0), 48.2% (95% CI: 42.9-53.3), 95.6% (95% CI: 91.598.1) and 64.1% (95% CI: 59.8-68.2), respectively.

In developing countries like Nepal, where hospitals with high dependency care are not accessible easily, HAPS can be used to identify patients whose disease course will be mild with minimal and substantial hospital costs in initial evaluation of patients which will prevent unnecessary referrals to tertiary centers. Our study was done in a single tertiary centre where most of the cases are referred from other centers where most of the AP patients had already had been resuscitated and referred which was the limitation of this study along with the small sample size. We recommend the need for validation of this severity assessment system at the community level in a multicenter setting involving a large sample size and including the clinical interventions done in hospital and their effect in the patient cohort.

## CONCLUSIONS

Our study showed HAPS to be a simple, attractive and promising algorithm consisting of only three parameters, namely signs of peritonitis or guarding, levels of serum creatinine and hematocrit, that is capable of reliably identifying the patients who will have a nonsevere course. The prevalence of harmless (harmless acute pancreatitis) among cases of acute pancreatitis was found to be similar to other studies. This helps in stratifying cases that can be managed in centres which lack high dependency units and thus minimizing the referrals to higher centers when not indicated. However, studies with the effect of hospital-based interventions on the clinical course of their patient in a large cohort are still to be reported. Until then, patients with predicted harmless disease based on the HAPS criteria should not be discharged home from the emergency room.
